# Risk Factors Associated with Blood Exposure for Sporadic Hepatitis E in Dhaka, Bangladesh

**DOI:** 10.4269/ajtmh.17-0261

**Published:** 2017-08-14

**Authors:** Hossain M. S. Sazzad, Stephen P. Luby, Alain B. Labrique, Saleem Kamili, Tonya M. Hayden, Nourine A. Kamili, Chong-Gee Teo, Emily S. Gurley

**Affiliations:** 1 icddr,b, Dhaka, Bangladesh;; 2 Division of Global Health Protection, Center for Global Health, Centers for Disease Control and Prevention (CDC), Atlanta, Georgia;; 3 Stanford University, Stanford, California;; 4 Johns Hopkins Bloomberg School of Public Health, Baltimore, Maryland;; 5 Division of Viral Hepatitis, Centers for Disease Control and Prevention, Atlanta, Georgia

## Abstract

Fecal contamination of drinking water is associated with large hepatitis E virus (HEV) outbreaks of genotypes 1 and 2 in endemic areas. Sporadic transmission of HEV genotypes 3 and 4 in high-income countries has been associated with exposure to blood and animal contact. The objective of the study was to identify the risk factors for hepatitis E and the genotype(s) causing sporadic hepatitis E in Dhaka, Bangladesh. We selected, from a diagnostic center in Dhaka between November 2008 and November 2009, cases presenting with jaundice and anti-HEV IgM antibodies and age-matched controls were defined as those with no history of jaundice and normal blood test results. Serum samples were tested for HEV RNA using real-time reverse transcriptase polymerase chain reaction followed by a sequencing and phylogenetic analysis. A total of 109 cases and 109 controls were enrolled. The cases were more likely to be male (adjusted matched odds ratios [mOR] 2.2, 95% CI: 1.2–3.9; *P* = 0.01), or have reported contact with another person’s blood or blood product, or contact with blood-contaminated sharp instruments (adjusted mOR 2.1, 95% CI: 1.1–4.1; *P* = 0.03) than controls. There were no significant differences between the cases and the controls in terms of reported high-risk sexual intercourse, consumption of undercooked meat, or contact or drinking fecally-contaminated water. The sera from three cases carried HEV RNA, all belonging to genotype 1. Findings from this study suggest that contact with human blood and sharing sharp instruments may transmit sporadic hepatitis E in Dhaka, Bangladesh. Efforts to prevent the transmission of blood-borne pathogens may also prevent sporadic HEV transmission in this endemic setting.

## INTRODUCTION

Hepatitis E is a common cause of enterically transmitted hepatitis worldwide.^1^ Drinking fecally-contaminated water is associated with large hepatitis E outbreaks in endemic areas, including the Indian subcontinent.^2,3^ Hepatitis E virus (HEV) genotypes 1 and 2 have been repeatedly identified in waterborne outbreaks.^4^ HEV infection is endemic in Bangladesh where occasional outbreaks are reported.^5–8^ Hepatitis E caused by HEV genotypes 3 or 4 is, by contrast, a zoonosis,^9,10^ which in high-income countries has been associated with consumption of raw deer meat,^11^ undercooked liver and meat from pigs and boars,^12–14^ and occupational exposure to pigs.^15,16^

Recent studies have suggested that in some circumstances HEV may be transmitted parenterally. In England, HEV infection has been identified among blood component recipients,^17^ though these tend to be associated with infection by HEV genotypes 3 or 4.^18–20^ It is unclear whether the genotypes 3 and 4 have been associated with this transmission route because they are the prevailing genotypes in these countries, or if they are more likely transmissible through parenteral routes compared with other genotypes. A case-control study from rural Bangladesh found that endemic hepatitis E cases were more likely to report exposure to blood-contaminated sharps than controls within the 3 months preceding the onset of the case’s illness.^21^ However, the genotype of HEV that caused infection was unknown. Transient viremia is a feature of acute HEV infection caused by all four HEV genotypes. As HEV viremia lasts about 2–3 weeks around the onset of illness,^22^ blood-borne transmission of HEV is possible.

In lower/middle-income settings like Bangladesh, there is no surveillance for hepatitis E; hence, the detection of hepatitis E cases and subsequent risk factor identification requires special studies. Risk factor identification of sporadic hepatitis E and subsequent prevention might reduce disease spread and burden. Knowledge of circulating HEV genotype(s) among sporadic HEV cases can inform vaccination strategies against the prevailing HEV genotype(s) in the community. The objective of the present study was to identify the risk factors for sporadic hepatitis E and the causative HEV genotype(s).

## MATERIALS AND METHODS

### Study setting.

Dhaka is a rapidly growing mega-city with a population of more than 12 million.^23^ The Popular Diagnostic Center is a private diagnostic laboratory in southwest Dhaka, with three additional branches serving as serum specimen collection centers for anti-HEV IgM testing. The laboratory tested around 10 patients per day for anti-HEV IgM antibodies using an enzyme-linked immunosorbent assay manufactured by the Diagnostic Systems (Saronno, Italy). Its diagnostic sensitivity was reported as 98% and specificity as 95.2%.^24^ The laboratory diagnosed hepatitis E when the signal to cut-off (S/Co) ratio at optical density 450 nm was > 1.2.

### Case recruitment.

From November 2008 through November 2009, the study team identified patients with acute jaundice with yellow sclera on the palms or skin of those who were referred to the Popular Diagnostic Center for anti-HEV IgM testing. We enrolled cases whose sera showed S/Co > 2.5 in the anti-HEV IgM assay. This ratio was higher than the standard 1.2 cutoff to minimize potential false positives. Eligible cases were individuals of any age and gender who had resided in Dhaka for at least 2 months before study enrollment and verbally consented to have their laboratory test results shared with our study team.

We excluded enrollment of cases where people reported someone with similar jaundice-related illness in their home, neighborhood, workplace, or place of study between the 2 months preceding the onset of illness of the case and the date of testing at the Popular Diagnostic Center. We also excluded cases presenting in clusters, defined as two or more laboratory-confirmed hepatitis E cases tested at the Popular Diagnostic Center who lived within the same *mouza* (smallest administrative unit in Bangladesh) who had the onset of illness within 2 months of each other, considering that the incubation period of hepatitis E is 15–60 days.^25^ We did not test cases for hepatitis C virus or other blood-borne viral infections.

### Control recruitment.

We enrolled controls from the laboratory who had sought a blood test at the Popular Diagnostic Center for job recruitment, international travel, or other administrative and excluded those who sought testing for any disease including acute jaundice and fever. We excluded those who reported a lifetime history of jaundice or had ever been diagnosed with laboratory-confirmed viral hepatitis. We also excluded the controls who reported that any of their family members had a history of jaundice or a laboratory-diagnosed viral hepatitis within 2 months of the matched case’s diagnosis of hepatitis E. We did not consider acute viral hepatitis cases without detectable IgM antibodies against HEV as controls because they might have been infected with another hepatitis virus or blood-borne virus that has overlapping risk factors with hepatitis E, including exposure to blood-contaminated sharps.

Every day, the study team reviewed the blood test results recorded at the Popular Diagnostic Center to identify patients tested for total count of white blood cell, differential count of white blood cell, erythrocyte sedimentation rate, and hemoglobin percentage with results that were within the normal range, so as to select those whose results suggested no infection.^26^ The study team asked the clients, as they came to the laboratory for their results, if they were living in Dhaka during the 2 months before the blood test, and if so, if they were interested in participating in the study. Those who agreed were enrolled as controls.

We selected one age-matched control for each case from the list using the predetermined 5-year age ranges. In addition, we selected controls who tested blood within 2 months of the onset of the matched case’s illness.

### Data collection.

We trained the field team to interview the cases and controls using a structured, pretested questionnaire in Bengali. At the time of enrollment, the study team recorded the household address of the study participants and scheduled an interview at the participants’ homes. During the interview, the study team asked the participants about their signs and symptoms of illness, if any, and asked the cases about their exposures during the 2 months preceding the onset of illness. Considering the incubation period of hepatitis E,^25^ the time frame of exposure was between 15 and 60 days before the onset of illness. For healthy controls, we asked about exposures in the 2 months preceding their blood test.

We asked all participants about exposures known to be associated with hepatitis E, which included contact with another person’s blood or with sharp instruments such as needles for therapeutic injections before the onset of illness or the barber’s razor. However, we did not ask whether syringes used for injections were observed to be opened from a new package or had been previously used. We specifically excluded the exposure to any injection or intravenous saline received during the current illness. Moreover, we asked all participants about other parenteral exposures, including recreational intravenous drug use, donating blood or blood products or receiving a transfusion, undergoing a dental treatment, having their face or armpits shaved at a barbershop, and undergoing a surgery. We also asked all participants if they had any contact with domestic animals and birds, including rats, consumption of undercooked meat, or drinking water likely to be unclean. We asked adult participants about direct oral contact with the anogenital region of their sex partners or indirect oral contact through possible fecally contaminated hands during anal sex in the 2 months before the onset of illness or visit to the laboratory.

### Data analysis.

We compared the sociodemographic characteristics of the cases and controls. To estimate the association between specific risk exposures and hepatitis E, we calculated the matched odds ratios (mOR) and 95% confidence intervals (CI) using a conditional logistic regression. We included variables in the model for the exposures that could be confounders, for example, gender.

We conducted a univariate analysis to explore the association between each specific exposure and hepatitis E. We considered *P* value ≤ 0.05 as a priori cutoff value for the level of significance. Then we combined some specific exposures post hoc into broader categories to increase statistical power to detect differences between the cases and controls. We pooled the exposure to therapeutic injections, contact with other people’s blood, undergoing dental treatment, being shaved at a barbershop, or undergoing a surgery into the post hoc combined category of exposure to blood. We pooled keeping domestic animals and having observed rodents or their excrement at home into the zoonotic post hoc combined category. We also pooled eating grilled meat or kebab into the post hoc combined zoonotic category. We pooled engagement in oral or anal sex into the post hoc combined sexual exposure category. All pooled post hoc combined exposure categories with associated *P* values of 0.2 or less in the univariate analysis were included in the multivariate conditional logistic regression model.^27^ We analyzed data in the STATA 13 (Stata Corporation, College Station, TX).

### Laboratory testing.

We stored an aliquot of serum from hepatitis E cases at −70°C. We recorded the date of sample collection and the date of the onset of illness for each case. We determined the number of days between the date of the onset of illness and the date of sample collection and identified a subset of cases whose samples were collected within 14 days of the onset of illness. We shipped an aliquot of serum from those cases to the Division of Viral Hepatitis laboratory at Centers for Disease Control and Prevention (CDC), Atlanta, for confirmatory testing of IgM and IgG antibodies against HEV using a commercial assay (DSI, Sarrono, Italy). Serum samples were tested for HEV RNA using a quantitative real-time reverse transcriptase polymerase chain reaction (PCR) assay targeting a 69-bp fragment of open reading frame (ORF) 3.^28,29^ The positive samples were subjected to another reverse transcriptase PCR targeting a 258-bp fragment of ORF 1 for the sequencing and phylogenetic analysis.^29,30^ The sensitivity and specificity of the PCR assay is 100%. We selected the samples collected within 14 days of the onset of illness for genotyping because viremia may be present for up to 2 weeks after the onset of jaundice.^31^ The phylogenetic tree was constructed from sequences from the cases and also from cases in recent hepatitis E outbreaks in Tongi^7^ and Rajshahi^32^ (both in Bangladesh) and other neighboring Asian countries. The phylogenic tree was created with a maximum parsimony, close neighbor-interchange algorithm, and 1,000 bootstrap replicates.^33^ Branch lengths were in units of the number of changes over the whole sequence.

### Human subject protection.

The study team sought verbal informed consent for reviewing the test results during preliminary enrollment from the study participants. Before case-control interviews, the team obtained informed written consent from study participants or their legal guardians if they were < 18 years of age. An assent form was also administered for participants 8–17 years old. The study protocol was approved by the Ethical Review Committee of icddr,b (Protocol number: 2007-056) and the Human Research Protection office of US CDC (Protocol number: 5323).

## RESULTS

### Case-control study.

Through the Popular Diagnostic Center, we approached 160 laboratory-confirmed hepatitis E cases. Among these cases, we excluded 31 cases because they were constituents of hepatitis E clusters. Among the remaining 129 hepatitis E cases, we could not match 20 cases with the controls. Of the 133 potential controls identified, 24 could not be matched with cases. Hence, a total of 109 cases and 109 age-matched controls were enrolled. Of the 109 cases, 108 (99%) reported loss of appetite, 107 (98%) had a new onset of yellow sclera, 106 (97%) reported dark urine, 104 (95%) had nausea or vomiting, 103 (94%) had extreme weakness, 94 (86%) had fever, 79 (72%) had pain in the right hypochondrium, and 69 (63%) reported passing ash- or clay-colored stool during the last illness.

The cases were more commonly male compared with the controls (65% versus 43%, *P* = 0.001). There was no difference with respect to age [mean, 29 years (range: 11–59) versus 29 (range: 10–58)], monthly household expenditure (> 130 US$: 86% versus 88%), or education (≥ 11 years: 97% versus 97%) between the cases and the age-matched controls (Table 1).

**Table 1:** Sociodemographic characteristics of sporadic hepatitis E cases and age-matched controls, Dhaka, Bangladesh, Nov 2008–Nov 2009

		Cases = 109 *n* (%)	Control = 109 *n* (%)	*P*
Age in years				
	< 15	7 (6%)	7 (6%)	1.00
	15–39	78 (72%)	78 (72%)	1.00
	≥ 40	24 (22%)	24 (22%)	1.00
Mean age in years (range)		29 (11–59)	29 (10–58)	1.00
Gender				
	Female	38 (35%)	62 (57%)	0.001*
	Male	71 (65%)	47 (43%)	0.001*
Marital status				
	Married	48 (44%)	59 (54%)	0.136
	Unmarried	59 (54%)	48 (44%)	0.136
	Widowed	2 (2%)	1 (1%)	0.56
Monthly expenditure of the household (US$)				
	≤ 80	8 (7%)	4 (4%)	0.235
	81–130	7 (6%)	9 (8%)	0.603
	> 130	94 (86%)	96 (88%)	0.686
Ownership				
	Electric fan	108 (99%)	108 (99%)	1.00
	Bicycle	0 (0%)	0 (0%)	undefined
	Mobile phone	107 (98%)	109 (100%)	0.155
	Radio	68 (62%)	73 (67%)	0.479
	Television	95 (87%)	99 (91%)	0.387
	Refrigerator	92 (84%)	91 (83%)	0.854
	Private vehicle	25 (23%)	27 (25%)	0.751
Highest level of education obtained among all family members				
	No schooling (0 years)	0 (0%)	0 (0%)	undefined
	Up to primary (1–5 years)	0 (0%)	1 (1%)	0.316
	Up to secondary (6–10 years)	3 (3%)	2 (2%)	0.651
	Above secondary (≥ 11 years)	106 (97%)	106 (97%)	1.00

* *P* value statistically significant.

No single exposure with associated *P* value ≤ 0.05 was associated with being a hepatitis E case in the age-matched univariate analysis (Table 2). The blood-borne exposures of the cases and controls included exposure to therapeutic injections, contact with other people’s blood, undergoing dental treatment, being face or armpit shaved at a barbershop, and undergoing surgery (Table 2). Demographic factors and post hoc combined (pooled) exposures with associated *P* values ≤ 0.2 in the univariate analysis included being male, contact with another person’s blood or sharing sharp instruments, animal contact in the home, and presence of stagnant water near the home (Table 3).

**Table 2:** Univariate analysis of single risk factors among sporadic hepatitis E cases and age-matched controls, Dhaka Bangladesh, Nov 2008–Nov 2009

Exposures within time frame*	No. and % of cases with this risk factor	No. and % of control with this risk factor	mOR and 95% CI	*P*
Blood exposure				
Any therapeutic injection or vaccine (excluding that given for current disease)	13 (12%)	11 (10%)	1.2 (0.5–2.9)	0.66
Number of injections or vaccines taken				
One time	8 (7%)	3 (3%)	3.5 (0.7–16.8)	0.12
Two or more times	5 (5%)	8 (7%)	0.5 (0.2–1.9)	0.60
IV saline infusion at least once	2 (15%)	0 (0%)	2 (0.2–22.1)	0.57
Recreational intravenous drug use	0 (0%)	0 (0%)	undefined	undefined
Blood donation	5 (5%)	2 (2%)	4 (0.4–35.8)	0.22
Blood or blood product transfusion	1 (1%)	1 (1%)	1 (0.1–15.9)	1.00
Shaving any part of body in barbershop	57 (52%)	45 (41%)	1.8 (0.9–3.2)	0.07
Touching other people’s blood or blood product or handling used syringe needle with bare hand	11 (10%)	5 (5%)	2.5 (0.8–7.9)	0.12
Zoonotic				
Observing rat or mice in home	53 (49%)	44 (40%)	1.5 (0.8–2.6)	0.20
Observing excrement of rat or mice in home	18 (17%)	22 (20%)	0.8 (0.4–1.6)	0.48
Eating meat				
Beef	91 (85%)	99 (91%)	0.6 (0.3–1.4)	0.24
Mutton	53 (50%	49 (45%)	1.2 (0.7–1.9)	0.59
Chicken	107 (100%)	108 (98%)	undefined	undefined
Pork	0 (0%)	0 (0%)	undefined	undefined
Eating undercooked meat†				
Beef	11 (20%)	10 (16%)	1 (0.3–3.9)	1.00
Mutton	0 (0%)	0 (0%)	undefined	undefined
Chicken curry	8 (8%)	8 (8%)	1.2 (0.4–3.5)	0.78
Pork	0 (0%)	0 (0%)	undefined	undefined
Chicken breast (fried meat)	55 (51%)	58 (53%)	0.9 (0.5–1.5)	0.69
Grilled meat	35 (32%)	44 (41%)	0.7 (0.4–1.2)	0.20
Kabab	71 (65%)	65 (60%)	1.3 (0.7–2.1)	0.42
Liver	63 (58%)	65 (60%)	0.9 (0.6–1.6)	0.79
Sexual				
Men and women who had receptive anal sex	3 (6%)	2 (4%)	0.6 (0.1–7.1)	0.69
Man who had insertive anal sex	0 (0%)	3 (6%)	0.5 (0.04–5.5)	0.57
Man and woman who had receptive oral sex	1 (2%)	1 (2%)	undefined	undefined
Man who had insertive oral sex	0 (0%)	1 (2%)	undefined	undefined
Exposure to water				
Drinks consumed outside home				
Commercially available water	71 (66%)	71 (65%)	1.1 (0.6–1.9)	0.77
Municipal water	24 (22%)	17 (16%)	1.7 (0.8–3.7)	0.18
Bottled mineral water	88 (83%)	80 (73%)	1.8 (0.8–3.5)	0.10
Boiled water brought from home	39 (36%)	36 (33%)	1.1 (0.6–1.9)	0.78
Fresh fruit juice	34 (31%)	31 (28%)	1.2 (0.6–2.1)	0.65
Cane juice with ice	15 (14%)	11 (10%)	1.6 (0.6–4.1)	0.35
Cane juice without ice	8 (7%)	6 (6%)	1.5 (0.4–54.3)	0.53
Lassi	38 (35%)	31 (28%)	1.4 (0.8–2.4)	0.31
Nature of drinking water inside home				
Boiled water	61 (56%)	66 (61%)	0.8 (0.5–1.4)	0.50
Untreated municipal water	5 (5%)	4 (4%)	1.3 (0.34–4.6)	0.73
Filtered water	41 (38%)	39 (36%)	1.1 (0.6–1.8)	0.79
Respondent observations about municipal water				
Turbidity or alteration of color	56 (51%)	57 (52%)	0.9 (0.6–1.7)	0.98
Odor	54 (50%)	41 (38%)	1.5 (0.9–2.5)	0.10
Dirty particle	54 (50%)	43 (39%)	1.4 (0.9–2.4)	0.16
Dirty skim	76 (70%)	70 (64%)	1.3 (0.7–2.4)	0.38
Stagnant water anywhere near home	53 (49%)	39 (36%)	1.8 (0.9–3.4)	0.06
Water stored in any large reservoir or water tank	108 (99%)	108 (99%)	1 (0.1–16)	1.00
Water tank was not cleaned	77 (71%)	70 (65%)	1.3 (0.7–2.4)	0.38
Water tank had any leak	4 (4%)	4 (4%)	1 (0.2–4.9)	1.00

* Exposures in the 2 months (excluding the recent 2 weeks) before the onset of illness of case and blood sample collection of healthy controls.

† Food was not cooked for optimum time and at temperature required to change the color, consistency, and flavor of meat.

**Table 3:** Univariate and multivariate analysis of post hoc combined risk factors among sporadic hepatitis E cases and age-matched controls, Dhaka Bangladesh, Nov 2008–2009

Exposures within time frame*	No. and % of cases with this risk factor	No. and % of controls with this risk factor	Univariate analysis		Multivariate analysis	
			mOR and 95% CI	*P*	Overall adjusted mOR and 95% CI	*P*
Demographic factor						
Male gender	71 (65%)	47 (43%)	2.3 (1.3–4.1)	0.003	2.2 (1.2–3.9)	0.01
Exposure to blood						
Contact with another person’s blood or blood product, or reported contact with blood contaminated sharp instruments†	70 (64%)	53 (49%)	2.2 (1.8–4.2)	0.014	2.1 (1.1–4.1)	0.03
Zoonotic						
Presence of domestic animal or observing rodents in the house†	59 (54%)	48 (44%)	1.6 (0.89–2.8)	0.12	1.6 (0.8–3)	0.15
Eating grilled meat/kebab/chicken broast†	84 (77%)	80 (73%)	1.2 (0.66–2.2)	0.547		
Sexual						
Oral contact with genitalia during sex†	3 (3%)	5 (5%)	0.6 (0.14–2.5)	0.48		
Exposure to water						
Consumed water from a source outside home	97 (89%)	93 (85%)	1.4 (0.6–2.9)	0.435		
Observed altered quality of municipal water	68 (62%)	64 (59%)	1.2 (0.68–2.01)	0.579		
Stagnant water anywhere near home	53 (49%)	39 (36%)	1.8 (0.9–3.4)	0.06	1.8 (0.9–3.5)	0.08
Mean duration of living in Dhaka city	12.2 years (9.9–14.5)	14.3 years (11.9–16.6)				

* Exposures in the 2 months (excluding the recent 2 weeks) before the onset of illness of case and blood sample collection of healthy controls.

† Post hoc combined exposures.

In the multivariate conditional logistic regression, the factors independently associated with hepatitis E were being male (adjusted mOR 2.2, 95% CI: 1.2–3.9; *P* = 0.01) and contact with another person’s blood or sharing sharp instruments (adjusted mOR 2.1, 95% CI: 1.1–4.1; *P* = 0.03) (Table 3). There were no significant differences reported between the cases and controls in terms ofhigh-risk sexual intercourse, contact with domestic animal, consumption of undercooked meat, or contact with or drinking fecally-contaminated water.

### Laboratory testing.

**Figure 1.**
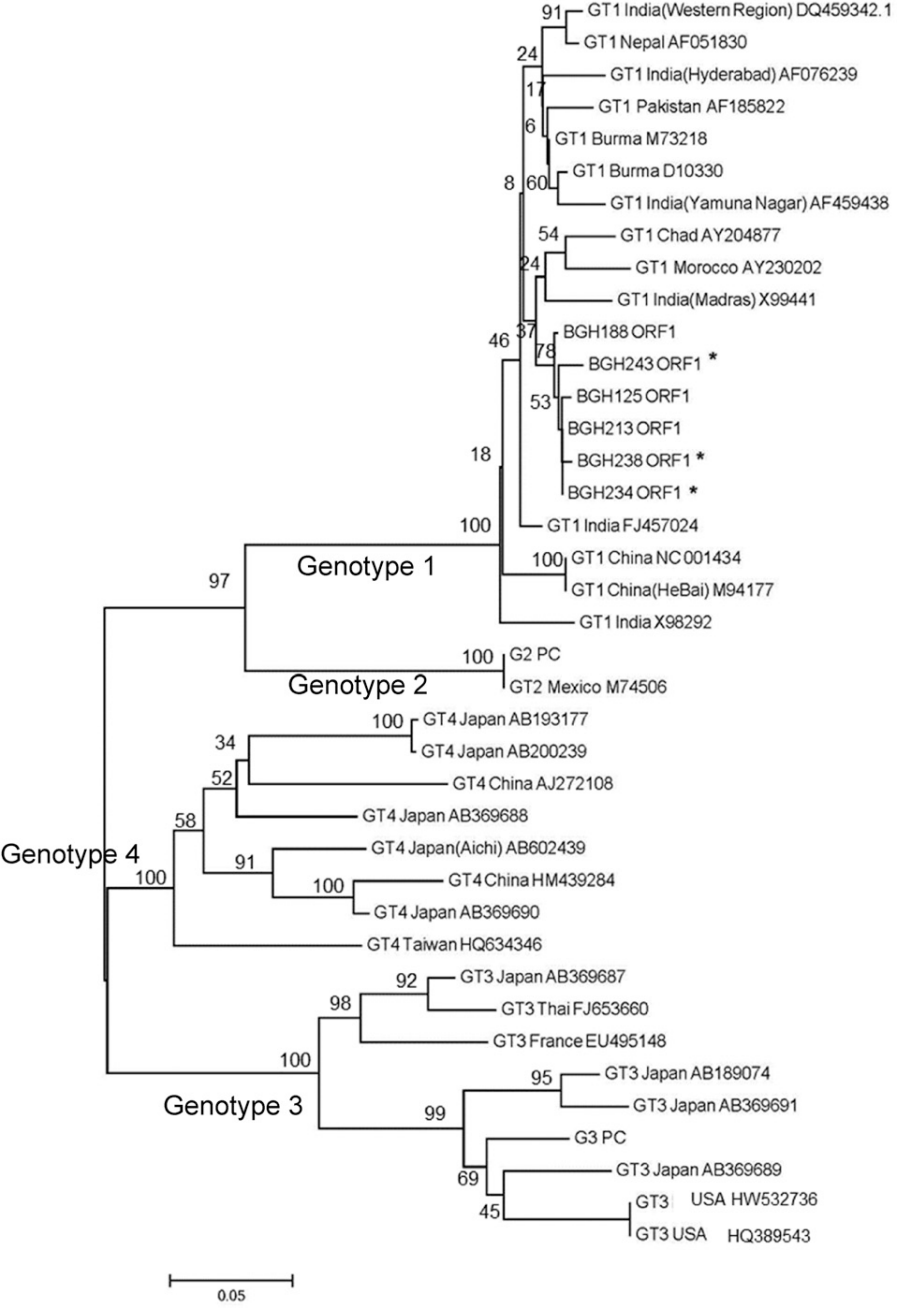
Phylogenetic analyses of sequences from the HEV ORF1. Sequences were trimmed to match the 238 bp region sequenced for the three positive samples. Available GenBank accession numbers are shown for corresponding sequences. BGH = Bangladesh; ORF = open reading frame. Scale bars indicate the number of sequence changes corresponding to the illustrated branch length.

All serum samples initially with detectable IgM antibodies against HEV were confirmed to have detectable IgM and IgG antibodies against HEV. Serum samples of 17 cases were collected within 14 days of the onset of illness. Serum samples from these 17 cases were tested for HEV RNA of which three (BGH234ORF1, BGH238ORF1, and BGH243ORF1) were positive. Sequencing showed that they belonged to the HEV genotype 1. These sequences clustered with those from previous outbreaks in Bangladesh and India (Figure 1).

### Exposure among cases with confirmed HEV genotype 1 infection.

Among the three genotype 1 confirmed cases, one case was a male laboratory worker. He had touched a laboratory client’s blood with his bare hands, donated blood for transfusion, and received a shave from a barbershop in the 2 months before the onset of illness. Among the two remaining cases, one male reported of eating kebabs in the 2 months before the onset of illness. Another female case reported of observing rodents in the house and eating grilled meat and kebab. All three cases reported visible dirt, altered color, and bad odor of their household supply water; however, they all reported that they usually boiled the supply water before drinking.

## DISCUSSION

Our study suggests that some sporadic hepatitis E cases in Dhaka may result from exposure to blood. Risk factors for sporadic hepatitis E in urban Dhaka were recent contact with other people’s blood, blood products, or exposure to other people’s blood through contact with sharp instruments during face or armpit shaving in the barbershop. Our data are consistent with other studies suggesting blood-borne HEV transmission. The blood-borne transmission of HEV was identified in a hospital setting in Pakistan through patients sharing intravenous administration sets.^34^ In addition, chronic hemodialysis, hemophilic patients,^35,36^ and individuals with a history of minor surgery^37^ were found to be at risk of hepatitis E. Patients with thrombotic thrombocytopenic purpura had serological and molecular evidence of HEV infection after a pooled plasma transfusion, suggesting plausible parenteral transmission of HEV.^38^ The HEV RNA has been identified in human blood from 1 to 42 days of the onset of illness.^39^ The HEV viremia may occur in asymptomatic adults in endemic areas.^40^ Therefore, individuals may become infected with HEV through contact with the blood of a hepatitis E patient or an asymptomatic individual during viremia through possible breached skin or mucosa, through the transfusion of blood containing HEV, through contact with instruments containing HEV-contaminated blood during face or armpit shaving from a barbershop, or through invasive medical or surgical procedures.

Among patients seeking primary health care from government subdistrict hospitals of Bangladesh, 78% of patients received an injection during treatment, and 42% of patients received IV infusion for nonspecific general weakness symptoms, consistent with the preference of the population to treatment with injections for quick relief.^41^ In low- and lower-middle income countries, it is possible that sharp instruments are reused at the point of service. For example, barbers may reuse blades,^42^ and health care providers may reuse injection equipment without taking effective steps to disinfect the equipment.^41^ Other hepatitis viruses like hepatitis C virus can survive in needle-syringes from 7 to 63 days.^43^ Subsequently, the HEV contaminated sharps may act as a vehicle for the transmission of HEV and other blood-borne pathogens to other persons. Similar unsafe injection practices were found in the health care facilities of other lower-middle-income countries including Pakistan^44^ and Egypt.^45^ Further research efforts should include better understanding of the practices and motivations for use, cleaning, and reuse of blood-contaminated sharp objects in Bangladesh.

In theory, the risk of blood-borne transmission of a specific genotype of HEV is higher for the genotype that is circulating among the population in an HEV-endemic area, assuming that there are no differences in infectivity through this route among genotypes. The HEV genotype 1 was found among the sporadic hepatitis E cases in urban Dhaka; it was consistent with the same genotype circulating among both outbreaks and sporadic cases in the Indian subcontinent. This study suggests that viremia resulting from acute infection by the HEV genotype 1 can facilitate parenteral HEV transmission.

Boiling drinking water at home is common and during 2011, 50% of the households of Dhaka district drank boiled, bottled, or filtered water at home.^23^ Our study participants had a higher economic status than most Dhaka residents so they likely had the resources to boil their water at home regularly. In our study, 56% (61/109) of the cases reported that they usually drank boiled water from home, and we asked about their usual practices, so sometimes they may not have boiled water at their home. Also, the water they drank outside the home was unlikely boiled.

The injection drug users are at risk of viral hepatitis B and C infections because hepatitis B and C viruses are transmitted parenterally from one person to another through injection needle sharing.^46^ High prevalence (17–23%) of the HEV infection has been identified among injection drug users in nonendemic high income countries.^47,48^ None of the enrolled cases reported taking injections for substance abuse, probably because they might be reluctant to report injection drug use. Research on prevalence and risk factors for the transmission of hepatitis E among the injection drug users in an endemic setting might further explore the possibility of blood-borne transmission of the HEV.

A limitation of the study was a reduction in power because of fewer than expected eligible controls visiting the private laboratory for blood tests. Moreover, our finding of an association between blood exposure and hepatitis E, although biologically plausible, should be interpreted cautiously, both because the effect size of the association was small and because the analysis that combined individual exposures to blood was conducted post hoc which could increase the likelihood that this association is biased or untrue.^49^

The enrolled cases were more likely to be male than the healthy controls, and an association of gender and hepatitis E was found. One possible explanation could be that we selected the study participants from a healthcare facility in Bangladesh, where males seek healthcare more than females.^50^ However, it is unlikely that this selection bias substantially affected our results because the multivariate conditional logistic regression model was adjusted for the effect of gender during the analysis.

Our study may not be generalizable to the whole population of Dhaka. During 2010, the average monthly per household expenditure of urban Bangladesh was US$120.^51^ Close to 90% of study participants reported > 130 US$ monthly household expenditure, suggesting that most study participants had a higher economic status than most Dhaka residents because they could afford the cost of serological testing from a private laboratory; this assumption was also supported by the fact that most participants attained more than secondary school education. Possible blood-borne transmission of HEV among our study participants of high socioeconomic status suggests that low income slum dwellers in Dhaka are also at risk of blood-borne transmission of HEV because they seek low-cost health care, where they may receive injection through recycled needle syringe and because they receive shaves in barbershops where sharing unclean razors is common.^52,53^

In conclusion, the sporadic hepatitis E cases in Dhaka tended to have been exposed to blood or blood-contaminated sharp instruments. HEV genotype 1, the primary circulating HEV genotype in this region, could be characterized from some cases. Efforts to prevent blood-borne infections, such as promotion of safe injections and using disposable blades at barbershops, may also reduce the risk of acquiring and spreading HEV infection.
